# Predictive and prognostic value of folate receptor-positive circulating tumor cells in small cell lung cancer patients treated with first-line chemotherapy

**DOI:** 10.18632/oncotarget.17039

**Published:** 2017-04-11

**Authors:** Jiqiao Shen, Jing Zhao, Tao Jiang, Xuefei Li, Chao Zhao, Chunxia Su, Caicun Zhou

**Affiliations:** ^1^ Department of Medical Oncology, Shanghai Pulmonary Hospital, Thoracic Cancer Institute, Tongji University School of Medicine, Shanghai, 200433, P.R. China; ^2^ Department of Lung Cancer and Immunology, Shanghai Pulmonary Hospital, Tongji University School of Medicine, Shanghai, 200433, P.R. China

**Keywords:** small cell lung cancer, circulating tumor cell, count

## Abstract

To assess the predictive and prognostic significance of folate receptor (FR)-positive circulating tumor cells (CTCs) in patients with small cell lung cancer (SCLC) received first-line chemotherapy. Eligible patients with chemotherapy-naïve, unresectable SCLC were enrolled and blood samples were collected. CTCs were enumerated using ligand-targeted polymerase chain reaction (LT-PCR) at baseline, after two cycles of chemotherapy regimen and on disease progression. In total, 80 patients were enrolled and 67 (83.8%) had positive CTC count at baseline (CTCs ≥ 8.7 FU/3mL). The baseline CTC counts in patients with partial response (PR) were significantly higher than those with progression disease (PD) (*P* = 0.0365). An obvious reduction of CTC enumeration after two cycles of chemotherapy was significantly correlated with PR (*P* = 0.0380), instead of SD (*P* = 0.4934). Among positive CTC count group, patients with relative low CTC level had significantly longer progression-free survival (PFS) and overall survival (OS) than those with high CTC level (PFS: 9.1 vs 6.9 months, *P* = 0.0458; OS: 11.1 vs 8.6 months, *P* = 0.056). In multivariate analysis, distant metastases (HR = 1.466, *P* = 0.021) and relative low CTC level (HR = 0.656, *P* = 0.049) were the independent predictive factors for patients with SCLC received first-line chemotherapy. The present results demonstrated that baseline CTC counts could be the valuable predictive and prognostic biomarker for patients with SCLC received first-line chemotherapy. The reduction of CTC enumeration after two cycles of chemotherapy was a potential predictor of chemotherapeutic response in SCLC.

## INTRODUCTION

Small cell lung cancer (SCLC) accounts for approximately 10–15% of all lung cancer cases and is a leading cause of cancer-related death worldwide [1, 2]. The majority of patients, about 60–70%, are diagnosed with extensive-stage disease and the overall prognosis remains dismal. Although SCLC is highly sensitive to chemotherapy and radiotherapy, development of drug resistance occurs frequently during the course of disease which leads to a high relapse rate [3]. Unfortunately, there is a lack of validated biomarkers for predicting the therapeutic efficacy of chemotherapy in patients with SCLC.

As a novel biomarker, circulating tumor cells (CTCs) has been proved to be a significant prognostic factor in metastatic prostate cancer, breast cancer, gastric cancer, colorectal cancer, cutaneous melanoma and non-SCLC (NSCLC) [4–9]. CTCs are cells that originate from the detachment of the primary or metastatic tumor mass and migrate into the circulatory system. CTC-based liquid biopsy provides a simple and convenient method for tracking cancer progression. Recently, several studies have demonstrated that CTCs could also predict the treatment response and prognosis in SCLC [10–16]. Hou et al. concluded from their study that baseline CTC count was the independent prognostic factor for both progression-free survival (PFS) and overall survival (OS) [10]. However, Chen et al. later reported a contrasting result, showed that baseline CTC enumeration was just the prognostic factor for OS, but not for PFS. Instead of CTC counts at baseline, CTC counts after two cycles of chemotherapy was a better indicator for predicting the prognosis of SCLC [14]. However, due to the difference in CTC detection method, non-standardized cutoff value and diverse treatment regimens of the previous studies, the predictive and prognostic role of CTC counts in SCLC remains controversial. In the afore-mentioned studies, CTC enumeration was performed by the CellSearch system (Veridex LLC, Raritan, NJ) approved by the U.S. Food and Drug Administration (FDA) for the prognosis in colorectal, breast and prostate cancer, but not in lung cancer. CellSearch is an immunomagnetic-based CTC enrichment system, which detects epithelial cell adhesion molecule (EpCAM)-positive CTCs by immunostaining. However, this system can not detect EpCAM-negative CTCs.

Folate receptor (FR)-positive CTCs which are shown to be highly expressed in lung cancer could be a possible alternative to predict SCLC prognosis [17]. Previous studies have established a novel method, named ligand-targeted polymerase chain reaction (LT-PCR) to detect FR-positive CTCs in patients with lung cancer [18–20]. With a cutoff value of 8.7 FU/3mL determined by ROC curve analysis, results revealed that FR-positive CTCs have a high sensitivity (72–76%) and specificity (82–88%) in the diagnosis of lung cancer, indicating that FR-positive CTCs had a promising clinical significance. Recently, the LT-PCR method used in this study has been approved by the China FDA (CFDA) for clinical application. Although this method has been demonstrated to be useful in the diagnosis of lung cancers, its application in predicting treatment response of SCLC requires further clarification. Therefore, in the present study, we aimed to investigate both the predictive and prognostic significance of FR-positive CTCs in SCLC patients treated with first-line chemotherapy.

## RESULTS

### Patient's characteristics

**Figure 1:**
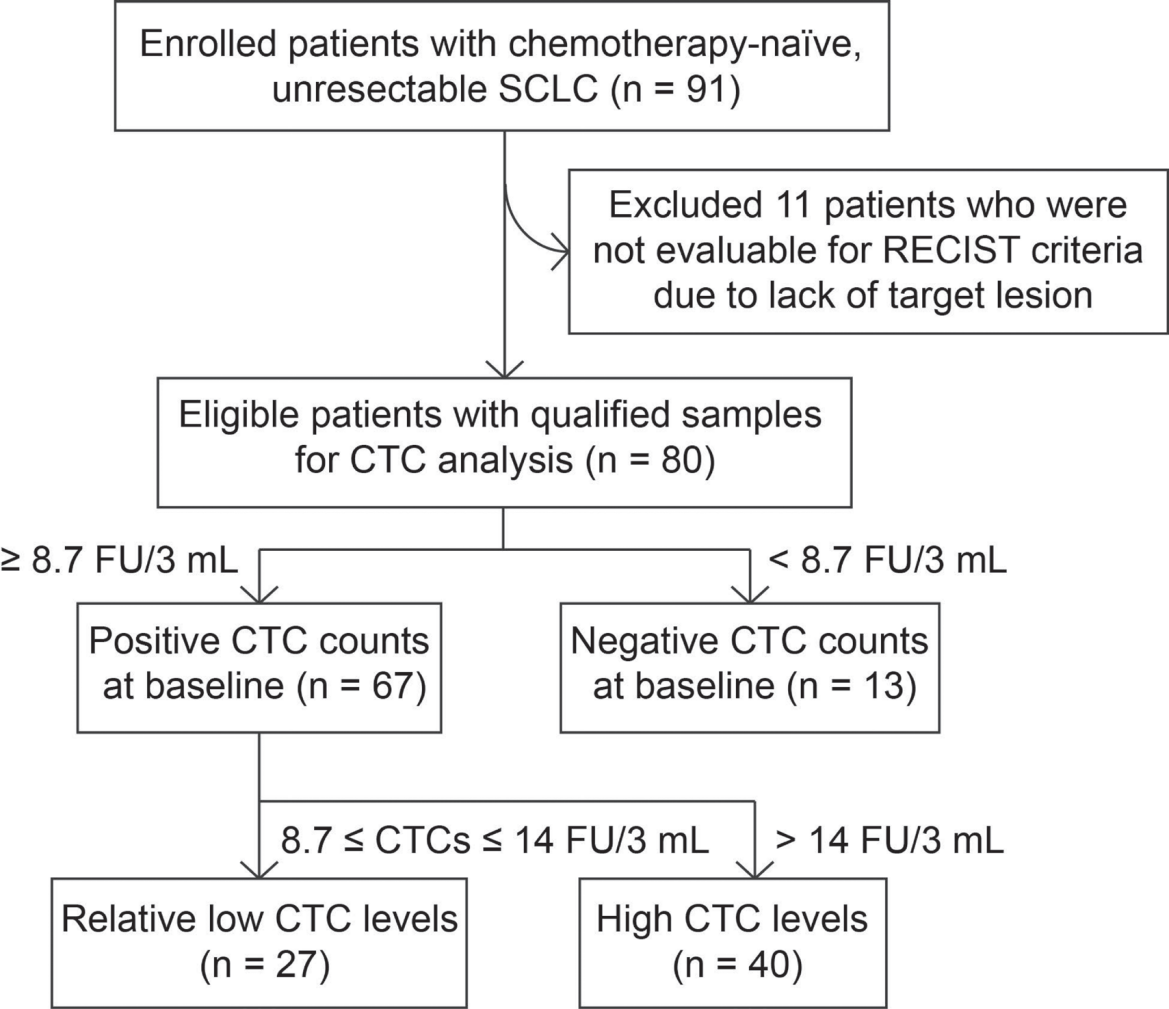
Flow chart of patient inclusion

In total, 91 consecutive patients with SCLC were included into the study between September 2014 and March 2016, of which 11 patients were not evaluable for RECIST criteria due to lack of target lesion. 80 patients with qualified samples were included into the CTC analysis. With 8.7 FU/3mL as the cutoff threshold, 67 of 80 patients (83.8%) had a positive CTC counts at baseline (Figure 1). The median baseline CTC value is 13.89 FU/3 mL. Patient demographics were listed in Table 1. There were no significant associations between baseline CTC counts and clinical characteristics including age, sex, smoking history, metastases and stage at diagnosis (Table 1).

**Table 1:** Clinical and molecular characteristics of included SCLC patients

	Total (*n* = 80)	CTC counts (/3 mL)	*P* value	CTC positive (*n* = 67)	CTC negative (*n* = 13)	*P* value
**Age (years)**						
Median	62 (41–81)			62 (41–81)	60 (49–75)	
< 65	51	16.9	0.310	41	10	0.794
≥ 65	29	14.7		24	5	
**Gender**						
Male	69	16.2	0.880	58	11	0.800
Female	11	15.7		9	2	
**Smoking history**						
Never-smoker	30	16.4	0.871	24	6	0.481
Former/current smoker	50	16.0		43	7	
**Metastases**						
Yes	40	18.0	0.113	35	5	0.363
No	40	14.3		32	8	
**Stage**						
I-II	4	14.0	0.523	3	1	0.181
III-IV	76	16.2		64	12	
VALG stage						
Limited stage	3	13.7	0.414	2	1	0.984
Extensive stage	77	16.4		65	12	
**Regimen**						
Etoposide+cisplatin	60	15.6	0.650	48	12	0.835
Etoposide+carboplatin	16	18.3		15	1	
Etoposide	4	15.5		4	0	

### Correlation between baseline CTC counts and radiological response

**Figure 2:**
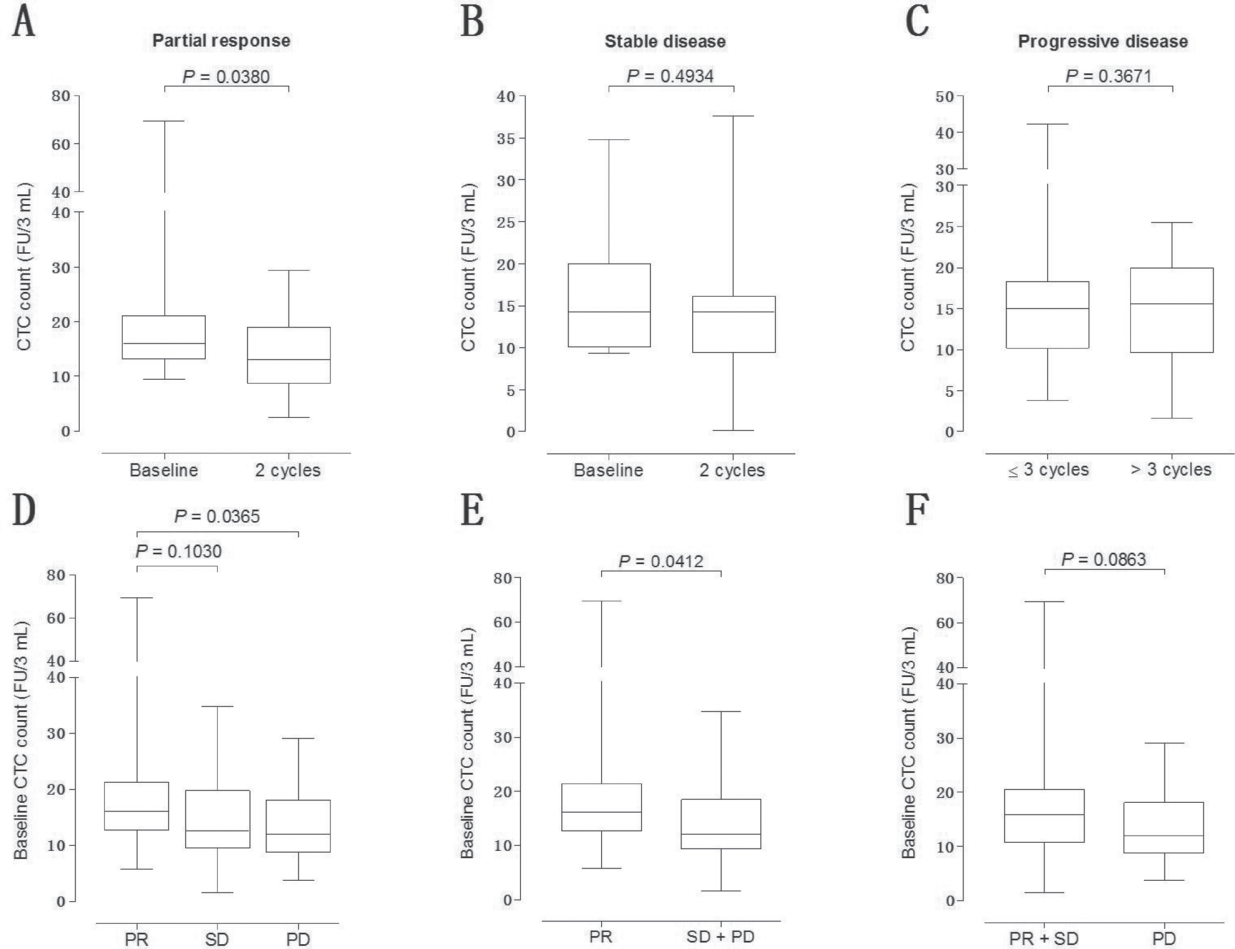
Relationship between baseline CTC counts and radiological response (**A**) CTC counts at baseline vs after two cycles of first-line chemotherapy in SCLC patients with PR; (**B**) CTC counts at baseline vs after two cycles of first-line chemotherapy in SCLC patients with SD; (**C**) CTC counts in patients acquired PD within vs after 3 cycles of chemotherapy; (**D**) comparison of baseline CTC counts in patients with PR vs SD vs PD; (**E**) comparison of baseline CTC counts in patients with PR vs SD+PD; (**F**) comparison of baseline CTC counts in patients with PR+SD vs PD.

To investigate the predictive significance of baseline CTC counts in first-line chemotherapy response, radiographic follow-up was performed by computer tomography (CT) scan according to RECIST criteria. After two cycles of chemotherapy, partial response (PR), stable disease (SD) and progressive disease (PD) were observed in 26, 18 and 15 patients, respectively (Table 2). To validate the association of the change in CTC level with the treatment response, we compared the CTC counts at baseline with that detected after two cycles of chemotherapy. For patients with PR, a significant decrease in CTC level was observed after two cycles of chemotherapy (*P* = 0.0380, Figure 2A). However, the change in CTC level for patients with SD was not significant (*P* = 0.4934, Figure 2B). To explore whether the baseline CTC counts can be used to predict the time of disease progression in SCLC, patients are divided into two groups according to the time when PD occurred. Patients acquired PD within three cycles of chemotherapy were divided into the ‘refractory disease group’, while PD occurred after three cycles of chemotherapy were divided into the ‘treatment-sensitive group’. No difference was observed between the baseline CTC counts of these two groups of patients (*P* = 0.3671, Figure 2C). Patients acquired PR after chemotherapy showed a significantly higher baseline CTC counts than patients with PD (*P* = 0.0365, Figure 2D), instead of SD (*P* = 0.1030, Figure 2D). Intriguingly, patients with PR or SD showed a significantly higher baseline CTC counts than patients with PD (*P* = 0.0412, Figure 2E) but patients with PR showed a marginally significantly higher baseline CTC counts than patients with SD or PD (*P* = 0.0863, Figure 2F).

**Table 2:** Comparison of the chemotherapy efficacy according to CTC counts

	Relative low CTC level (*n*=27)	High CTC level (*n*=40)	*P* value
**Radiological response**			
Complete response	0	0	
Partial response	11	15	
Stable disease	8	10	
Progressive disease	4	11	
Undefined	4	4	
**Clinical response rate**			
Objective response rate	11 (40.7%)	15 (37.5%)	0.969
Disease control rate	19 (70.4%)	25 (62.5%)	0.506

### Relationship between baseline CTC counts and objective response

To assess the relationship between CTC count at baseline and objective response to first-line chemotherapy, we examined 80 patients and their radiological data in this study. At baseline, both the objective response rate (ORR) and disease control rate (DCR) of CTC positive patients were similar to that of CTC negative patients (ORR: 38.8% vs 30.8%, *P* = 0.814; DCR: 65.7% vs 53.8%, *P* = 0.417). To further explore the optimal cutoff value of CTC counts, patients with positive CTC counts were divided into two groups according to their baseline CTC level. CTC counts between 8.7 and 14.0 FU/3 mL were defined as the relative low CTC level group and CTC counts higher than 14.0 FU/3mL was defined as the high CTC level group. Although the ORR and DCR of the relative low CTC level group were higher than that in the high CTC level group, it did not reach statistical significance (ORR: 40.7% vs. 37.5%, *P* = 0.969; DCR: 70.4% vs. 62.5%, *P* = 0.506, Table 2).

### Predictive significance of baseline CTC counts on PFS

**Figure 3:**
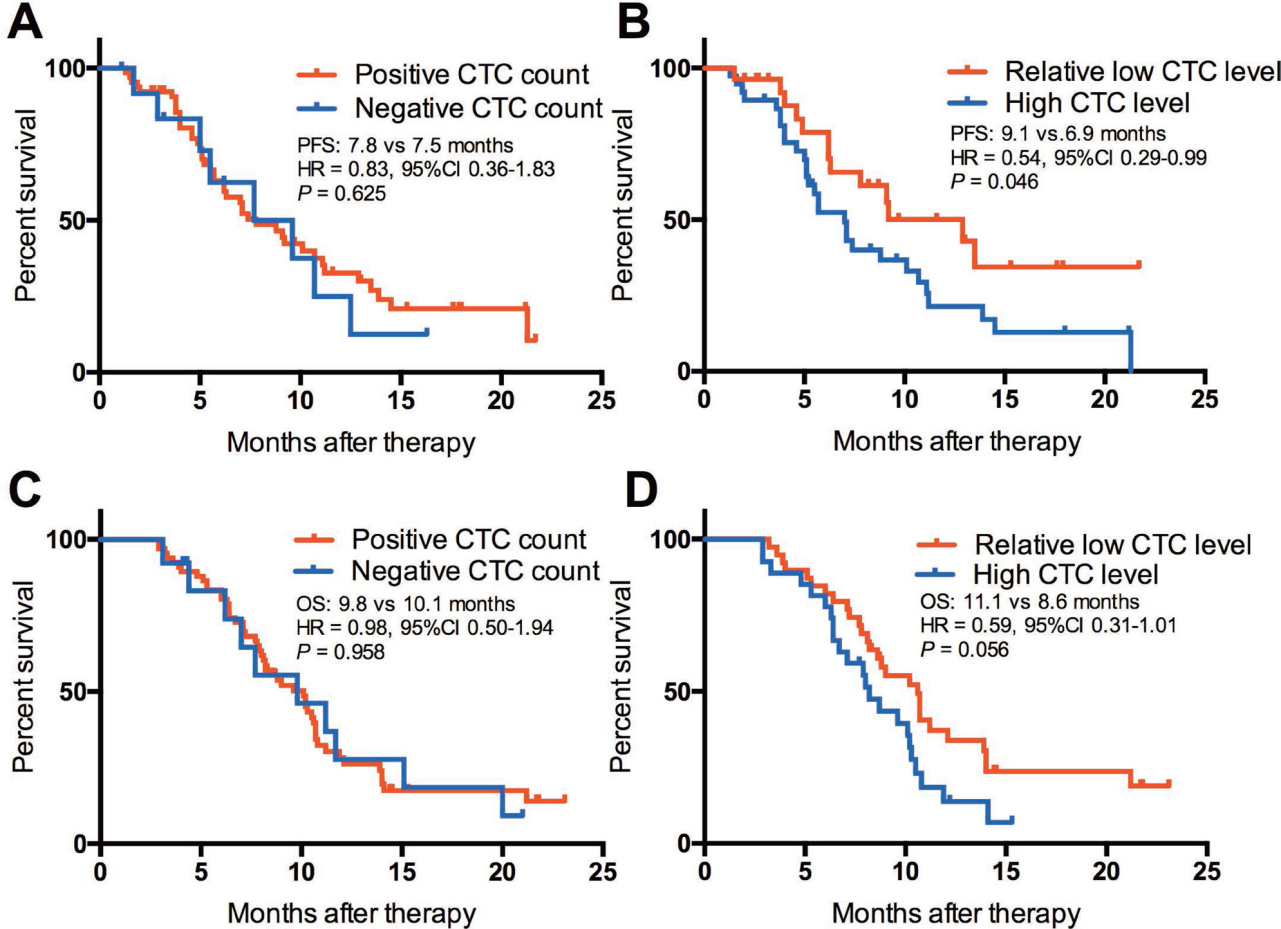
Kaplan-Meier curve of PFS and OS in SCLC patients (**A**) PFS of positive CTC level vs negative CTC level; (**B**) PFS of the relative low CTC level group vs the high CTC level group; (**C**) OS of positive CTC level vs negative CTC level; (**D**) OS of the relative low CTC level group vs the high CTC level group.

The median PFS of the patients with positive CTC count and negative CTC count at baseline had similar PFS (7.8 vs 7.5 months, HR = 0.83, 95% CI 0.36–1.83, *P* = 0.625, Figure 3A). When patients with positive CTC counts were divided into two groups according to their baseline CTC level, we found that patients with relative low CTC level had significantly longer PFS than those with high CTC level (9.1 vs 6.9 months, HR = 0.54, 95% CI 0.29–0.99, *P* = 0.0458, Figure 3B). In multivariate analysis, only distant metastases (HR = 1.466, 95% CI 1.071–2.554, *P* = 0.021) and relative low CTC level (HR = 0.656, 95% CI 0.473–1.000, *P* = 0.049) were the independent predictive factors for PFS in SCLC patients treated with first-line chemotherapy (Table 3).

**Table 3:** Multivariate analyses of clinical features on PFS and OS

Variable	PFS			OS		
	HR	95% CI	*P*	HR	95% CI	*P*
Age: < 65 vs. ≥ 65	0.704	0.561–1.319	0.473	0.961	0.695–1.172	0.395
Sex: male vs. female	1.217	0.877–1.954	0.861	1.096	0.827–2.311	0.482
Distant metastases: Yes vs. No	1.466	1.071–2.554	0.021	1.915	1.096–2.751	0.038
Smoking: never vs. current/former	0.727	0.486–1.579	0.162	0.841	0.614–1.897	0.531
CTC count: positive vs. negative	0.875	0.639–1.572	0.243	0.807	0.624–1.995	0.427
CTC count: relative low level vs. high level	0.656	0.473–1.000	0.049	0.715	0.551–1.821	0.139

Abbreviations: CTC, circulating tumor cell; HR, hazard ratio; CI, confidence interval.

### Prognostic value of baseline CTC counts on OS

We further investigated the prognostic value of CTC counts on OS in these patients. The results showed that patients with positive CTC count had similar OS when compared with patients with negative CTC count (9.8 vs 10.1 months, HR = 0.98, 95% CI 0.50–1.94, *P* = 0.958, Figure 3C). However, patients with relative low CTC level had marginally statistically significant longer OS than those with high CTC level (11.1 vs 8.6 months, HR = 0.59, 95% CI 0.31–1.01, *P* = 0.056, Figure 3D). In multivariate analysis, only distant metastases (HR = 1.915, 95% CI 1.096–2.751, *P* = 0.038) showed the prognostic value factors for OS in SCLC patients treated with first-line chemotherapy (Table 3).

## DISCUSSION

SCLC is an aggressive form of cancer which possesses a rapid disease progression and early spread of distant metastases. Currently, limited targeted therapy is available for SCLC treatment and chemotherapy remains the standard care of first-line treatment. Due to the aggressiveness of SCLC, a sensitive biomarker is urgently warranted to monitor the therapeutic effects in order to instantly and precisely provide evidence of disease progression and guide the follow-up treatment decision. Radiological technique such as CT scan is usually applied for monitoring the progress of SCLC. However, the association between radiological results and prognosis of SCLC patients remains uncertain [21]. Moreover, repeated CT scan could be detrimental to advanced-stage patients due to radiological overexposure and mental anguish. Therefore, a simple and non-invasive test, such as CTC enumeration, that could predict therapeutic response within a short period of time would be valuable. To our knowledge, the current study was the first to report both the predictive and prognostic significance of FR-positive CTCs in patients with SCLC, using a CFDA approved LT-PCR method. Comparing to conventional EpCAM-dependent CTC analysis, the negative enrichment procedure enables the collection of a wider variety of CTCs. Especially for malignant tumors, the majority of CTC population has undergone epithelial-mesenchymal transition (EMT) which lacks EpCAM expression and could lead to false negative results in EpCAM-dependent analysis [18, 22]. Additionally, through capturing of FR-positive CTCs using conjugates of a folic acid and a synthesized oligonucleotide, quantitative PCR would be performed subsequently which allows the precise quantification of CTC number.

With the reliable technique, the current study suggested that patients with SCLC had a high positive CTC counts rate (83.8%). Consistent with our results, several previous studies also indicated that SCLC is a highly malignant tumor possessing a high level of CTCs [10, 23]. There were no significant associations between the baseline CTC counts and the clinical characteristics including age, sex, smoking history, metastases and stage at diagnosis. However, Hou et al. reported that higher baseline CTC counts were significantly associated with number of metastases and liver metastases [10]. This discrepancy may attribute to the different detection methods and cutoff values between our and Hou et al.'s study (CellSearch). Theoretically, high baseline CTC counts increase the possibility of distant metastases. Hence, the relationship between baseline CTC counts and clinical characteristics, especially metastases, needs further validation.

The predictive value of baseline CTC counts in SCLC patients receiving chemotherapy has been investigated in several studies, but the results remain controversial. Cheng et al. reported that median PFS was similar between patient groups with ≥ 10 CTCs per 7.5 mL and < 10 CTCs per 7.5 mL at baseline [24]. This result was consistent with a recent study that indicated detection of CTCs was not associated with response to chemotherapy in SCLC [25]. However, Hou et al. reported that patients with low baseline CTC counts had significantly longer PFS than those with high baseline CTC counts (PFS: 8.8 vs 4.6 months, *P* < 0.001) [10]. Notably, all of these studies utilized CellSearch to enrich CTCs. As we mentioned previously, CellSearch system is dependent on EpCAM to capture CTCs from the blood. It would miss the representative CTC populations with EMT characteristics. In our study, we utilized FR as the target to label the CTCs and detected them using LT-PCR method. With the cutoff value of 14 FU/3mL, the present study showed that patients with relative low CTC level had dramatically longer PFS than those with high CTC level (CTCs ≥ 14), suggesting that high CTC counts are associated with worse PFS. Furthermore, our results also showed that baseline FR-positive CTC counts in patients with PR were significantly higher than those with PD, but no significant difference was observed between patients with PR and SD. The current evidence suggested that baseline CTC counts could be a potential predictor for the effect of first-line chemotherapy in patient with SCLC.

In addition to the baseline CTC enumeration, the dynamic change in CTC counts is also a significant predictor of treatment response. Cheng et al. reported that both the PFS and OS of patients with a drop in CTCs to < 10 per 7.5 mL after the second cycle of chemotherapy were longer than those with CTC count increased to ≥ 10 per 7.5 mL after treatment [24]. In the current study, we also found that patients with PR after two cycles of chemotherapy had an obvious reduction of CTC enumeration. This further demonstrated that change in CTC level is useful for predicting treatment response in SCLC.

The prognostic value of CTC counts in SCLC also have been extensively studied. In a multicenter prospective study, Hiltermann et al. found that Lack of measurable CTCs (27% of patients) was associated with prolonged survival (HR 3.4; *P* ≤ 0.001). CTC count after the first cycle of chemotherapy was the strongest predictor for overall survival (HR 5.7; *P* = 0.004) [12]. At the same time, another group also reported lower CTC levels were associated with a favorable outcome [26]. However, in 2014, Normanno et al. suggested that previously reported CTC cutoffs were not prognostic in their cohort of patients and only the change of CTC count after the first cycle of chemotherapy had the useful prognostic role in extensive SCLC [11]. Interestingly, our study found that detection of CTC was not a prognostic factor for OS but high CTC level showed the prognostic significance only in the patients with positive CTC counts. These inconsistent results indicated that whether the numeration of CTCs was the independent prognostic factor for OS in SCLC patients treated with first-line chemotherapy need the future investigation.

Our study had several limitations that should be well acknowledged. Firstly, the number of patients enrolled was relatively small. Secondly, not all of the eligible patient received the standard etoposide plus platinum regimen. Four of them received etoposide monotherapy due to poor performance status. Thirdly, the cutoff value of CTCs was derived from a single-institution cohort and was not validated by an independent validation cohort. A prospective study with a larger sample size is necessary for implementing our findings to routine clinical practice.

In conclusion, our study suggested that baseline FR-positive CTC counts and dynamic change in CTC enumeration were potential predictors of response to first-line chemotherapy in SCLC. FR-positive CTCs would play a crucial role in predicting the effect of first-line chemotherapy in SCLC patients and its value warrants further validation in a larger study.

## MATERIALS AND METHODS

### Study design

This was a prospective, single-institution clinical study conducted at Shanghai Pulmonary Hospital, Shanghai, China (trial registered number: ChiCTR-DDT-12003034). Patients with chemotherapy-naïve, histologically or cytologically confirmed SCLC were enrolled. Radiologically confirmed unresectable disease or operation intolerance was required, and patients were treated with standard EP (etoposide 100 mg/m2 d1-3, cisplatin 75 mg/m2 d1) or EC (etoposide 100 mg/m2 d1-3, carboplatin AUC = 5 d1) regimens. Prior palliative radiotherapy to the primary tumor or to a single site of metastasis, including brain metastasis, was permitted if 4 weeks before study inclusion. None of the patients had a history of prior malignancy within 5 years of study entry. The study was approved by the ethics committees of Shanghai Pulmonary Hospital and an informed consent was obtained from all participants. Peripheral blood samples were collected for CTC analysis within 1 days before receiving treatment (defined as the baseline), following two cycles of chemotherapy and when disease progressed. The major clinicopathological characteristics including demographic information, Eastern Cooperative Oncology Group performance status (ECOG PS), smoking history, lung cancer histology (WHO classification), metastatic status and treatment received were collected. A never smoker was defined as a person who had smoked less than 100 cigarettes during his lifetime. Smoking status, ECOG PS and age were documented at the time of diagnosis. Clinical outcomes were evaluated in accordance with the requirements established by Thoracic Cancer Institute, Tongji University School of Medicine.

### CTC analysis

CTC analysis was conducted using CytoploRare method provided by GenoSaber Biotech Co. Ltd. (Shanghai, China) as previously described [18–20]. Blood samples (3 mL) from enrolled individuals were collected in 5 ml EDTA anticoagulant tubes before commencing treatment, stored in 4°C refrigerator, and CTC analysis was conducted within 24 hours of collection, according to the manufacturer's instructions. Briefly, CTCs were enriched from 3mL of whole blood by immunomagnetic depletion of leukocytes and then labeled with conjugates of a tumor-specific ligand folic acid and a synthesized oligonucleotide. After washing off free conjugates, the stripped bound conjugates were analyzed by quantitative PCR. In this study, the quantity of CTC was expressed as an arbitrarily defined FR unit (FU), which was defined as the number of FR-positive CTCs tested in 3 mL of blood. A serial of standards containing oligonucleotides (10–14 to 10–19 M, corresponding to 2 to 2 × 10^5^ FU/3mL) were used for CTC quantification.

### Statistical analysis

The categorical variables were compared using chi-square tests, or Fisher's exact tests when needed. PFS was defined as the time from the date of first-line therapy initiation to the date of systemic progression or death and was censored at the date of last tumor assessment (when carried out). Disease progression was defined in accordance with the Response Evaluation Criteria in Solid Tumors (RECIST) version 1.1. Kaplan-Meier curve and two-sided log-rank test were used for univariate survival analyses. *P* values < 0.05 was considered to indicate a statistically significant difference. Statistical analysis was conducted with SPSS 18.0 software (SPSS Inc, Chicago, IL, USA) or the Prism 5.0 (GraphPad Software Inc, San Diego, CA, USA).
